# High-dose methylprednisolone pulse therapy during refractory COVID-19 acute respiratory distress syndrome: a retrospective observational study

**DOI:** 10.1186/s12890-023-02664-5

**Published:** 2023-10-03

**Authors:** Carsten Zeiner, Malte Schröder, Selina Metzner, Johannes Herrmann, Quirin Notz, Sebastian Hottenrott, Daniel Röder, Patrick Meybohm, Philipp M. Lepper, Christopher Lotz

**Affiliations:** 1 Department of Internal Medicine V, Saarland University Hospital, Kirrbergerstr. 100, 66421 Homburg/Saar, Germany; 2 Department of Anaesthesiology, Intensive Care, Emergency and Pain Medicine, University Hospital Würzburg, Oberduerrbacherstr. 6, 97080 Würzburg, Germany

**Keywords:** Corticoid, Methylprednisolone, Pulse therapy, SARS-CoV2, ECMO, Salvage Therapy

## Abstract

**Background:**

Current COVID-19 guidelines recommend the early use of systemic corticoids for COVID-19 acute respiratory distress syndrome (ARDS). It remains unknown if high-dose methylprednisolone pulse therapy (MPT) ameliorates refractory COVID-19 ARDS after many days of mechanical ventilation or rapid deterioration with or without extracorporeal membrane oxygenation (ECMO).

**Methods:**

This is a retrospective observational study. Consecutive patients with COVID-19 ARDS treated with a parenteral high-dose methylprednisolone pulse therapy at the intensive care units (ICU) of two University Hospitals between January 1st 2021 and November 30st 2022 were included. Clinical data collection was at ICU admission, start of MPT, 3-, 10- and 14-days post MPT.

**Results:**

Thirty-seven patients (mean age 55 ± 12 years) were included in the study. MPT started at a mean of 17 ± 12 days after mechanical ventilation. Nineteen patients (54%) received ECMO support when commencing MPT. Mean p_a_O_2_/F_i_O_2_ significantly improved 3- (p = 0.034) and 10 days (p = 0.0313) post MPT. The same applied to the necessary F_i_O_2_ 10 days after MPT (p = 0.0240). There were no serious infectious complications. Twenty-four patients (65%) survived to ICU discharge, including 13 out of 20 (65%) needing ECMO support.

**Conclusions:**

Late administration of high-dose MPT in a critical subset of refractory COVID-19 ARDS patients improved respiratory function and was associated with a higher-than-expected survival of 65%. These data suggest that high-dose MPT may be a viable salvage therapy in refractory COVID-19 ARDS.

**Supplementary Information:**

The online version contains supplementary material available at 10.1186/s12890-023-02664-5.

## Background

Moderate to severe COVID-19 induced acute respiratory syndrome (ARDS) is associated with high fatality [1, 2]. Standards of care include mechanical ventilation, prone positioning and in case of refractory hypoxemia extracorporeal membrane oxygenation (ECMO) [3]. A mainstay of supportive COVID-19 ARDS therapy is the administration of dexamethasone as an anti-inflammatory agent, reducing overall mortality [4]. German [3] and international guidelines [5] recommend its use. However, dexamethasone may not be the only glucocorticoid helping to ameliorate the outcome of COVID-19. Early administration of a medium dose methylprednisolone pulse improved the outcome in non-intubated COVID-19 patients [6], although without additional benefits to dexamethasone [7].

It remains unknown if high-dose methylprednisolone pulse therapy (MPT) ameliorates refractory COVID-19 ARDS after many days of mechanical ventilation and if needed ECMO. Supraphysiological quantities of methylprednisolone could help to reduce proinflammatory mediators, cells migrating into the lung and stop diffuse alveolar damage. To this extent high-dose methylprednisolone pulses, mostly administered over the course of three days, are common in acute exacerbations of idiopathic pulmonary fibrosis [8, 9] and nonspecific interstitial pneumonia [10]. Moreover, methylprednisolone has been associated with improved pulmonary compliance in non-COVID ARDS patients presenting with ECMO weaning failure [11]. However, evidence remains scarce and patient selection as well as toxicity, in particular infectious complications, may be of concern.

The current study aimed to assess the effects of salvage MPT on pulmonary function and outcome of COVID-19 ARDS.

## Methods

### Study design and patient population

This is a retrospective observational study. Consecutive patients with COVID-19 ARDS receiving a parenteral high-dose methylprednisolone pulse therapy (1000 mg/d) over the course of three days during their ICU stay were included in the study. Patients received MPT in case of refractory COVID-19, if two intensivists considered it to be indicated. All patients were treated at the University Hospital Würzburg or the Saarland University hospital between January 1st 2021 and November 30st 2022.

### Data collection and variable definition

Routine clinical data were collected at time of ICU admission, start of MPT, 3-, 10- and 14-days post MPT, as well as survival to ICU discharge using patient data management systems (University Hospital Würzburg: COPRA6 RM1.0, COPRA System GmbH, Berlin, Germany) or assessed via handwritten records (Saarland University Hospital). The data were retrieved according to the diagnostic standards of the individual center. Severe acute respiratory syndrome-coronavirus 2 (SARS-CoV-2) subtypes were not routinely determined. Mechanical power (MP) was calculated as follows: MP = 0.098.RR.*V*_*t*_'.[PEEP + Δ*Δ*_insp_] [12]. The term refractory COVID-19 relates to refractory hypoxemia in COVID-19 ARDS, defined as persistent or worsening hypoxemia unresponsive to lung protective ventilation. All patients included in the study had a p_i_O_2_/F_i_O_2_≤150, F_i_O_2_≥50% and/or concomitant ECMO support. Since treatment and data acquisition were conducted according to the standard procedures of the respective hospital, diagnostics and reported parameters varied to some degree between the centers. Hence, if applicable the nominators and denominators are reported for each parameter separately, since not all parameters could be retrieved in the whole cohort of patients. All participating hospitals reported their data via a unified sheet (Microsoft® Excel 2019, Version 16.41, Microsoft® Corporation, Redmond, WA).

### Statistical analysis

Pooled individual data reported as mean ± SD for continuous variables and n-numbers as well as frequencies for categorical variables. A mixed-effects model for repeated measures with post-hoc Tukey testing analysed differences of the overall study population. Mixed-effects analysis for multiple comparisons with post-hoc Šídák’s multiple comparisons test tested differences between groups. Prism 9.1.1 for macOS performed the data analysis. Statistical significance was determined at an α level of 0.05.

## Results

### Patient characteristics

Table 1 shows the characteristics of the 37 patients included in the retrospective analysis. Mean age at ICU admission was 55 ± 12 years; mean body mass index was 29 ± 5 kg/m^2^. MPT started 16 ± 12 days after ICU admission and 17 ± 12 days after starting mechanical ventilation. Seven patients received MPT < 10 days after ICU admission due to rapid deterioration. ECMO support was required in 21 patients with a mean duration of 40 ± 30 days. MPT commenced 11 ± 9 days after the initiation of ECMO. Seventeen patients had prone positioning. MPT was the only corticoid regime in 9 patients, whereas 15 patients received two different regimens and 13 patients had three or more different corticoids. In 21 patients, dexamethasone (in a dose of 6 mg once daily for up to 10 days [13] and MPT were the only two-corticoids. None of the patients had glucocorticoids as a long-term medication within the past six months. Four patients received tocilizumab (19, 16, 5 and 37 days prior to MPT, respectively). One patient received casirivimab and imdevimab in addition to tocilizumab.

**Table 1:** Patient Characteristics, ICU Treatment and Outcome

		Methylprednisolone Pulse Therapy
*Age [years]*		55 ± 12.2
*Sex m/f n [%]*		25/12 [68/32]
*BMI [kg/m* ^*2*^ *]*		29 ± 5.1
*Comorbidities n [%]*		
	Arterial Hypertension	13 [35]
	Coronary Artery Disease	4 [11]
	Diabetes mellitus	8 [22]
	Chronic obstructive pulmonary disease	1 [3]
	Chronic Renal Failure	0 [0]
*SAPS II Admission*		40 ± 18.1
*SAPS II Maximum*		54 ± 19
*Mechanical Ventilation [days]*		40 ± 31
*vvECMO Therapy n [%]*		21 [57]
*Duration vvECMO [days]*		40 ± 29.5
*Duration ICU Treatment [days]*		42 ± 34
*Survival n [%]*		24 [65]

BMI: Body Mass Index; SAPS II: Simplified Acute Physiology Score II; vvECMO: Venovenous Extracorporeal Membrane Oxygenation; ICU: Intensive Care Unit

### Respiratory function and mechanical ventilation

Mean p_a_O_2_/F_i_O_2_ was 110 ± 54 at ICU admission and 116 ± 44 at the start of MPT. Nineteen patients (54%) received ECMO support when commencing MPT. Mean p_a_O_2_/F_i_O_2_ significantly improved 3- (p = 0.034) and 10 days (p = 0.0313) post MPT. The same applied to the F_i_O_2_, which was significantly lower 10 days after MPT (p = 0.024). Mean tidal volume, driving pressure and mechanical power did not change. Serum C-reactive protein was significantly lower 3 days post MPT compared to ICU-admission (p = 0.0071) as well as to the start of MPT (p = 0.0056). ECMO support stopped in four patients (20%) within 10 days after MPT. When comparing survivors to non-survivors mean p_a_O_2_/F_i_O_2_ only improved in survivors (164 ± 69) 3 days post MPT, but not in non-survivors (115 ± 34) (p = 0.0569). F_i_O_2_ was 57 ± 23% in survivors and 76 ± 19% in non-survivors 3 days post MPT. Table 2 shows the characteristics of mechanical ventilation and respiratory mechanics of all patients receiving MPT. Severity of ARDS or F_i_O_2_ did not differ between survivors and non-survivors at ICU admission or start of MPT (Table S1). Characteristics of mechanical ventilation and respiratory mechanics in patients on ECMO support are shown in Table S2.

**Table 2:** Respiratory Function

Parameter		ICU-Admission (N = 37)	MPT (N = 37)	3 days post MPT (N = 32)	10 days post MPT (n = 24)	14 days post MPT (n = 22)	p-value
*F*_*i*_ *O*_*2*_ *[%]*		77 ± 19	77 ± 22	62 ± 23	58 ± 26# (p = 0.024)	67 ± 25 (N = 19)	0.007
*p*_*a*_ *O*_*2*_ */F*_*i*_ *O*_*2*_		110 ± 54	116 ± 44	151 ± 65* (p = 0.034)	151 ± 60* (p = 0.031)	149 ± 64 (N = 21)	0.006
*Tidal Volume [ml]*		480 ± 251 (N = 24)	335 ± 259 (N = 24)	403 ± 219 (N = 21)	326 ± 172 (N = 19)	294 ± 175§ (N = 15)	0.044
*Driving Pressure [cmH*_*2*_ *O]*		11 ± 8 (N = 24)	13 ± 5 (N = 24)	13 ± 6 (N = 20)	15 ± 5 (N = 15)	15 ± 5 (N = 11)	0.202
*Compliance* *[ml/cmH*_*2*_ *O]*		29 ± 29 (N = 21)	21 ± 24 (N = 23)	31 ± 38 (N = 17)	16 ± 10 (N = 13)	15 ± 12 (N = 10)	0.112
*Mechanical Power [J/min]*		30 ± 27 (N = 24)	20 ± 14(N = 24)	23 ± 14 (N = 19)	18 ± 13 (N = 12)	16 ± 15 (N = 10)	0.193
*CRP [mg/dl]*		8.6 ± 9.6	5.7 ± 7.3	2.9 ± 4.0*^#^ (*p = 0.005; ^#^p = 0.007)	6.1 ± 7.7	5.5 ± 5.7	0.002
*Ventilation Mode n [%]*	Spontaneous Breathing	0	1 (3)	0	5 (21)	6 (27)	0.001
	O_2_-Insufflation	3 (8)	0	2 (6)	0	1 (5)	
	High-Flow O_2_	10 (27)	12 (32)	9 (28)	4 (17)	4 (18)	
	PSV	5 (14)	9 (24)	10 (31)	6 (25)	2 (9)	
	PCV	18 (49)	14 (44)	11 (34)	8 (33)	8 (36)	
	APRV	1 (3)	1 (3)	0	1 (4)	1 (5)	
	ECMO	2 (5)	19 (54)	18 (51)	16 (66)	15 (68)	

*Significantly different vs. MPT; ^#^ significantly different vs. Admission; ^§^ significantly different vs. 3 days post MPT

ICU: Intensive Care Unit; MPT: Methylprednisolone Pulse Therapy; F_i_O_2_: Fraction of inspired oxygen; paO2: Arterial Partial Pressure of Oxygen; CRP: C-reactive Protein; PSV: Pressure Support Ventilation; PCV: Pressure Control Ventilation; APRV: Airway Pressure Release Ventilation; ECMO: Extracorporeal Membrane Oxygenation

### Outcome

Mean duration of ICU treatment was 42 ± 34 days. Mean duration of mechanical ventilation was 40 ± 31 days. Twelve patients had infectious complications, none of which resulted in septic shock. There were no other serious complications related to MPT, such as electrolyte disorders. Twenty-four patients (65%) survived to ICU discharge, including 13 out of 20 patients (65%) needing ECMO support.

## Discussion

MPT was a salvage therapy due to COVID-19 ARDS unresponsive to standard therapy or concomitant ECMO. Our study cohort received prolonged ICU care with a mean stay of more than 40 days. Prolonged ICU care is often defined as > 21 days [14] and associated with increased mortality with each day beyond seven days [15]. On average MPT started after more than two weeks of mechanical ventilation and if applicable eleven days of ECMO support. Although mean F_i_O_2_ and p_a_O_2_/F_i_O_2_ ratio were unchanged between ICU admission and start of MPT, native pulmonary function deteriorated as in the meantime half of the patients required ECMO support. As such, our study cohort represents a critical subset of COVID-19 ARDS patients with an expected survival in the range of 31.4% found in the German COVID-19 ECMO population [2]. Actual survival was much higher with 65% in both ECMO and non-ECMO patients. This is in accordance with a previously published cohort without prolonged ICU care [16] and is in range of a retrospective German reimbursement data analysis [17].

Prior studies investigating methylprednisolone in non-COVID-19 ARDS found varying results. Low dose prolonged infusions for up to 14–28 days starting in early ARDS had favorable effects on pulmonary function and duration of mechanical ventilation [18]. On the contrary, the administration of low dose methylprednisolone more than two weeks after the onset of on average moderate ARDS increased the risk of death [19]. Prior data on MPT in COVID-19 ARDS patients are limited and investigated a lower severity of illness. A recent observational retrospective study found an improved 28-day mortality in mechanically ventilated patients after high-dose MPT without significant increase in steroid-associated complications [20]. A lower dose MPT in severe COVID-19 requiring ICU treatment but not mechanical ventilation led to a significantly lower mortality [6]. In moderate COVID-19 MPT (250–500 mg/d for 3 days followed by oral prednisone for 14 days) lowered the need for ICU treatment and mortality [21]. A nationwide clinical cohort study in Japan showed that high-dose MPT was associated with a lower risk of in-hospital mortality in patients receiving mechanical ventilation but increased the risk in patients not receiving mechanical ventilation. Moreover, benefits of pulse therapy were higher than intermediate doses [22].

We observed positive effects of short-term high-dose MPT onto respiratory function after more than two weeks of mechanical ventilation. P_a_O_2_/F_i_O_2_ ratio significantly improved 3 days after the completion of MPT. The required F_i_O_2_ was also lower with significant effects 10 days post MPT. When comparing survivors to non-survivors it was apparent that non-survivors had no improvement in p_a_O_2_/F_i_O_2_ ratio or F_i_O_2_. ECMO weaning was not possible, whereas 25% of survivors needing ECMO completed weaning within 10 days and 50% within 14 days. Only 25% of survivors had ECMO 14 days post MPT compared to 75% of non-survivors. These data suggest that non-survivors may concomitantly be MPT non-responders. Side effects of high dose of methylprednisolone such as infectious complications and electrolyte disorders could be of concern, however, supraphysiological quantities of methylprednisolone are supposed to enhance therapeutic effects, while reducing toxicity. We did not observe severe complications. There were no septic complications or electrolyte disorders associated with MPT. Although an unspecific marker, serum C-reactive protein decreased, likely reflecting the immunosuppressive effects.

Our study has several limitations. It is only a small retrospective observational study. We cannot prove a survival benefit from MPT. The study cannot delineate MPT responders from non-responders. Our data do not allow the delineation of the underlying mechanisms or differences in SARS-CoV-2 subtypes. Sustained (hyper-)inflammatory injury due to dysregulation of many components of the innate immune system may be the responsible, followed by development of pulmonary fibrosis [23, 24]. Due to the uncontrolled design, use of any additional corticoid was at the discretion of the respective intensivists and the majority received dexamethasone as an additional glucocorticoid, in line with current German treatment guidelines [3]. We did not include a propensity score matched control group, as a sufficient number of matching patients with a similar protracted course was not available in our hospitals. Moreover, time points for the analysis of changes in respiratory function or laboratory parameters would not be available in propensity-matched controls, as data comparisons followed the start and course of MPT. These limitations reduce the validity of the results and additional evidence from randomized studies could control these biases.

## Conclusions

Our data suggest that short-term high-dose MPT can be a viable therapeutic option in refractory COVID-19 ARDS with persistent hypoxemia after prolonged mechanical ventilation and failure of ECMO weaning.

### Electronic supplementary material

Below is the link to the electronic supplementary material.

Supplementary Material 1

Supplementary Material 2

## Data Availability

The datasets used and/or analyzed during the current study are available from the corresponding author on reasonable request.

## Abbreviations

ARDS: Acute Respiratory Distress Syndrome
COVID-19: Coronavirus disease 2019
ECMO: Extracorporeal Membrane Oxygenation
ICU: Intensive Care Unit
MPT: Methylprednisolone Pulse Therapy
F_i_O_2_: Fraction of inspired oxygen
p_a_O_2_: Arterial partial pressure of oxygen
