# Supplementary material for: High-dose methylprednisolone pulse therapy during refractory COVID-19 acute respiratory distress syndrome: a retrospective observational study
Source: BMC Pulm Med. 2023 Oct 3;23:368. doi: 10.1186/s12890-023-02664-5 (PMC10546709; doi:10.1186/s12890-023-02664-5)
Supplement: Supplementary file 1 — Supplementary Material 1 [file 12890_2023_2664_MOESM1_ESM.docx]

**Table S1**. Respiratory Function comparing survivors and non-survivors

| Parameter |  | ICU-Admission | | Methylprednisolone Pulse Therapy (MPT) | | 3 days post MPT | | 10 days post MPT | | 14 days post MPT | | p-value |
| --- | --- | --- | --- | --- | --- | --- | --- | --- | --- | --- | --- | --- |
|  |  | *Survivor*  *(N=24)* | *Non-Survivor (N=13)* | *Survivor (N=24)* | *Non-Survivor (N=13)* | *Survivor (N=23)* | *Non-Survivor (N=9)* | *Survivor (N=16)* | *Non-Survivor (N=9)* | *Survivor (N=15)* | *Non-Survivor (N=8)* |  |
| *F_i_O_2_ [%]* |  | 75±21 | 80±12 | 71±19 | 78±21 | 57±23 | 76±19 | 56±26 (N=15) | 61±27 | 63±28 (N=11) | 73±21 | 0.0165 |
| *p_a_O_2_/F_i_O_2_* |  | 117±63 | 99±28 | 122±49 | 105±35 | 164±69*  (p=0.0569) | 115±34 | 158±77 (N=15) | 138±29 | 165±74 (N=13) | 121±30 | 0.0232 |
| *Tidal Volume [ml]* |  | 547±269 (N=15) | 368±179 (N=9) | 282±194 (N=13) | 313±167 (N=11) | 439±201 (N=13) | 345±250 (N=8) | 355±172(N=12) | 281±174 (N=8) | 310±163 (N=8) | 276±198 (N=7) | 0.0734 |
| *Compliance*  *[ml/cmH_2_O]* |  | 36±35 (N=13) | 18±9 (N=8) | 17±9 (N=12) | 24±33 (N=11) | 25±12 (N=9) | 37±55 (N=8) | 15±11 (N=7) | 16±8 (N=6) | 15±7 (N=5) | 16±16 (N=5) | 0.1979 |
| *Mechanical Power [J/min]* |  | 38±31 (N=15) | 18±8 (N=9) | 18±15 (N=13) | 22±10 (N=11) | 21±16 (N=12) | 21±13 (N=7) | 18±14 (N=8) | 18±12 (N=4) | 15±18 (N=5) | 16±14 (N=5) | 0.2573 |
| *CRP [mg/dl]* |  | 85±95 | 90±98 | 52±59 | 66±98 | 21±25 | 48±62 (N=9) | 60±87 | 64±56 (N=7) | 40±41 | 83±75 | 0.0180 |
| *Ventilation Mode n [%]* | Spontaneous Breathing | 0 | 0 | 0 | 1 (8) | 0 | 0 | 4 (25) | 1 (11) | 5 (33) | 1 (13) |  |
|  | O_2_-Insufflation | 2 (8) | 1 (8) | 0 | 0 | 2 (9) | 0 | 0 | 0 | 1 (7) | 0 |  |
|  | High-Flow O_2_ | 7 (29) | 3 (23) | 10 (42) | 2 (15) | 8 (35) | 1 (11) | 3 (19) | 1 (11) | 3 (20) | 1 (13) |  |
|  | PSV | 3 (13) | 2 (15) | 4 (17) | 5 (38) | 5 (22) | 5 (56) | 3 (19) | 3 (33) | 1 (7) | 1 (13) |  |
|  | PCV | 11 (46) | 7 (54) | 10 (42) | 4 (31) | 8 (35) | 3 (33) | 5 (31) | 3 (33) | 4 (27) | 4 (50) |  |
|  | APRV | 1 (4) | 0 | 0 | 1 (8) | 0 | 0 | 0 | 1 (11) | 0 | 1 (13) |  |
|  | ECMO | 2 (8) | 1 | 12 (50) | 7 (53) | 12 (52) | 6 (66) | 9 (56) | 6 (66) | 6 (40) | 6 (75) |  |

*Significantly different vs Non-Survivor; ICU: Intensive Care Unit; MPT: Methylprednisolone Pulse Therapy; F_i_O_2_: Fraction of Inspired Oxygen; p_a_O_2_: Arterial Partial Pressure of Oxygen; CRP: C-reactive Protein; PSV: Pressure Support Ventilation; PCV: Pressure Control Ventilation; APRV: Airway Pressure Release Ventilation; ECMO: Extracorporeal Membrane Oxygenation
