# Supplementary material for: High-dose methylprednisolone pulse therapy during refractory COVID-19 acute respiratory distress syndrome: a retrospective observational study
Source: BMC Pulm Med. 2023 Oct 3;23:368. doi: 10.1186/s12890-023-02664-5 (PMC10546709; doi:10.1186/s12890-023-02664-5)
Supplement: Supplementary file 2 — Supplementary Material 2 [file 12890_2023_2664_MOESM2_ESM.docx]

**Table S2:** Respiratory Function in Patients with ECMO Support

| Parameter | ICU-Admission  (N=21) | MPT (N=21) | 3 days post MPT (N=20) | 10 days post MPT (N=19) | 14 days post MPT (N=19) | p-value |
| --- | --- | --- | --- | --- | --- | --- |
| *F_i_O_2_ [%]* | 82±15 | 71±24 | 56±23  (^#^p=0.064) | 54±2(N=18)  (^#^p=0.019) | 67±25 (N=18) | 0.0001 |
| *p_a_O_2_/F_i_O_2_* | 99±37 | 124±53 | 164±74  (^#^p=0.011) | 156±66  (^#^p=0.01) | 158±63  (N=18,  #p=0.03) | 0.0002 |
| *Tidal Volume [ml]* | 484±274 (N=19) | 256±178 (N=18) (^#^p=0.02) | 367±204 (N=18)  (*p=0.034) | 305±175 (N=17) | 272±158 (N=14) | 0.0112 |
| *Driving Pressure [cmH_2_O]* | 14±7 (N=17) | 13±5 (N=18) | 14±5 (N=17) | 15±5 (N=12) | 15±5 (N=10) | 0.6605 |
| *Compliance*  *[ml/cmH_2_O]* | 29±32 (N=17) | 15±8 (N=17) | 21±12 (N=14) | 15±10 (N=12) | 12±7  (N=9) | 0.1249 |
| *Mechanical Power [J/min]* | 32±30  (N=19) | 16±14  (N=18) | 20±14 (N=16) | 18±14 (N=10) | 13±13 (N=9) | 0.1289 |
| *CRP [mg/dl]* | 10.5±11 | 6.7±9.4 | 3.7±4.8 | 6.8±8.3  (N=17) | 5.1±5.6 | 0.0403 |

*Significantly different vs. MPT; # significantly different vs. Admission

ICU: Intensive Care Unit; MPT: Methylprednisolone Pulse Therapy; FiO2: Fraction of inspired oxygen; paO2: Arterial Partial Pressure of Oxygen; CRP: C-reactive Protein; ECMO: Extracorporeal Membrane Oxygenation
